# Supplementary material for: On the correlation between material-induced cell shape and phenotypical response of human mesenchymal stem cells
Source: Sci Rep. 2020 Nov 4;10:18988. doi: 10.1038/s41598-020-76019-z (PMC7642380; doi:10.1038/s41598-020-76019-z)
Supplement: Supplementary file 1 — Supplementary Figures. [file 41598_2020_76019_MOESM1_ESM.pptx]

## Slide 1
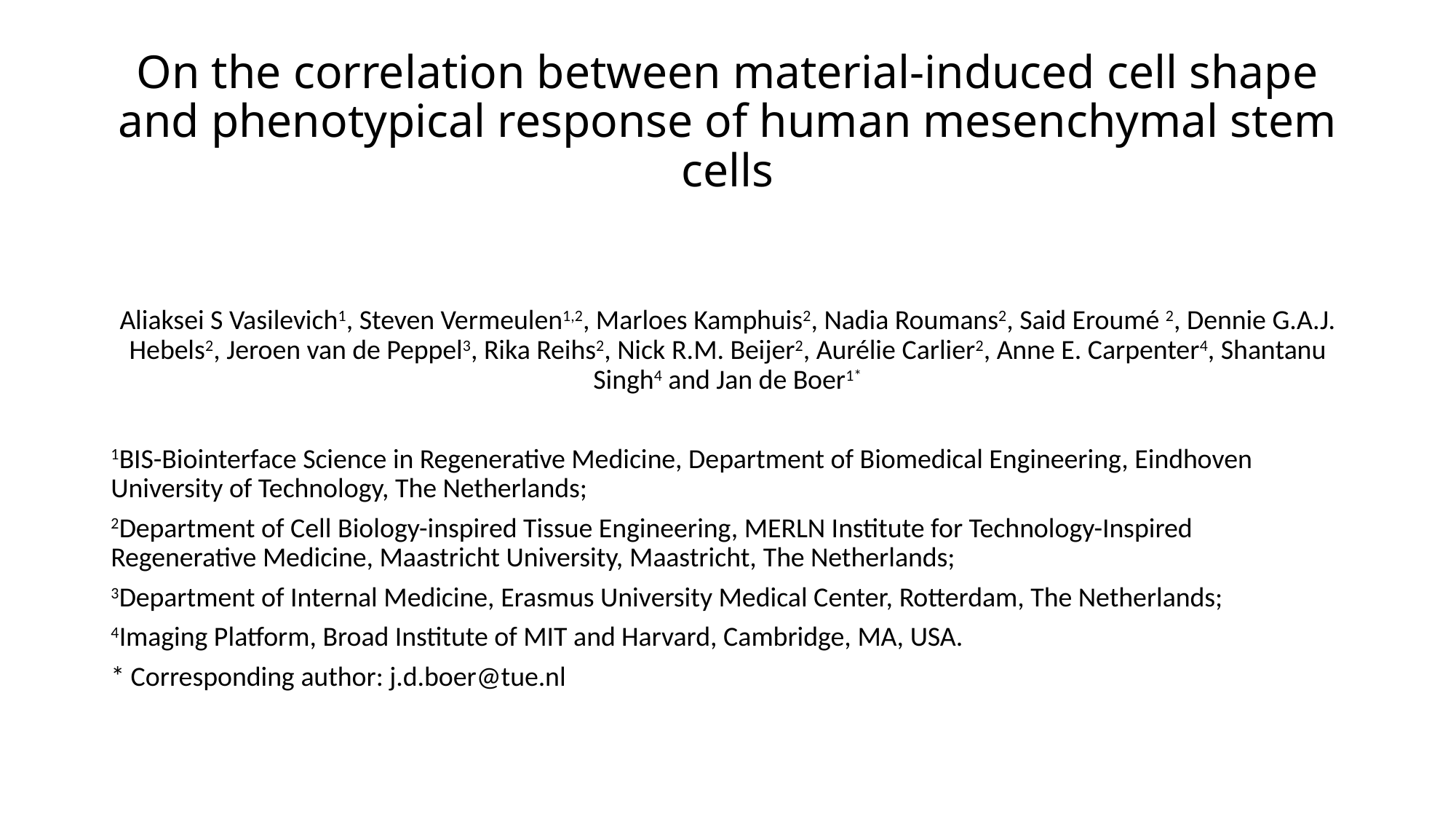

# On the correlation between material-induced cell shape and phenotypical response of human mesenchymal stem cells
Aliaksei S Vasilevich1, Steven Vermeulen1,2, Marloes Kamphuis2, Nadia Roumans2, Said Eroumé 2, Dennie G.A.J. Hebels2, Jeroen van de Peppel3, Rika Reihs2, Nick R.M. Beijer2, Aurélie Carlier2, Anne E. Carpenter4, Shantanu Singh4 and Jan de Boer1*
1BIS-Biointerface Science in Regenerative Medicine, Department of Biomedical Engineering, Eindhoven University of Technology, The Netherlands;
2Department of Cell Biology-inspired Tissue Engineering, MERLN Institute for Technology-Inspired Regenerative Medicine, Maastricht University, Maastricht, The Netherlands;
3Department of Internal Medicine, Erasmus University Medical Center, Rotterdam, The Netherlands;
4Imaging Platform, Broad Institute of MIT and Harvard, Cambridge, MA, USA.
* Corresponding author: j.d.boer@tue.nl

## Slide 2
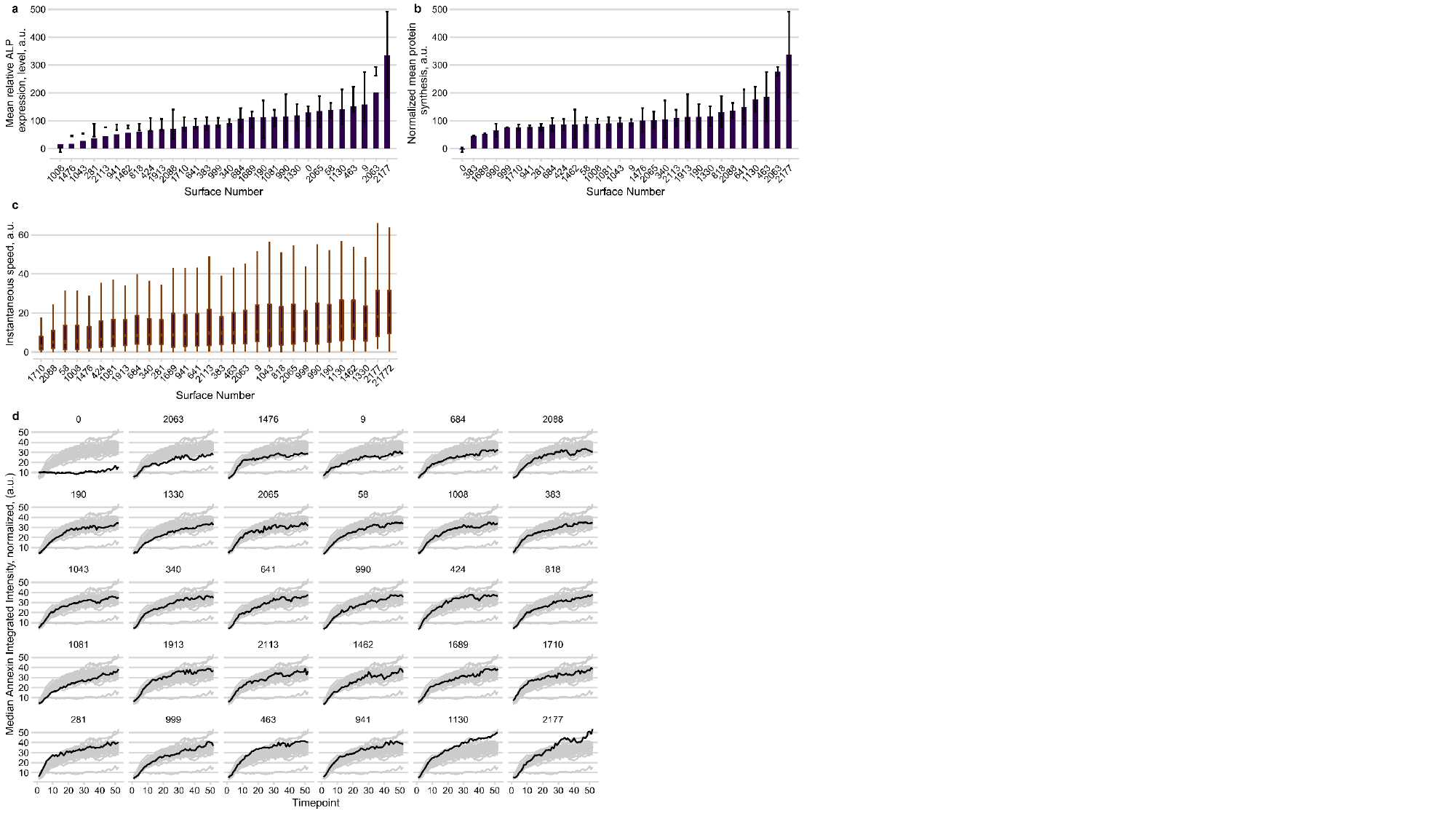

#

## Slide 3
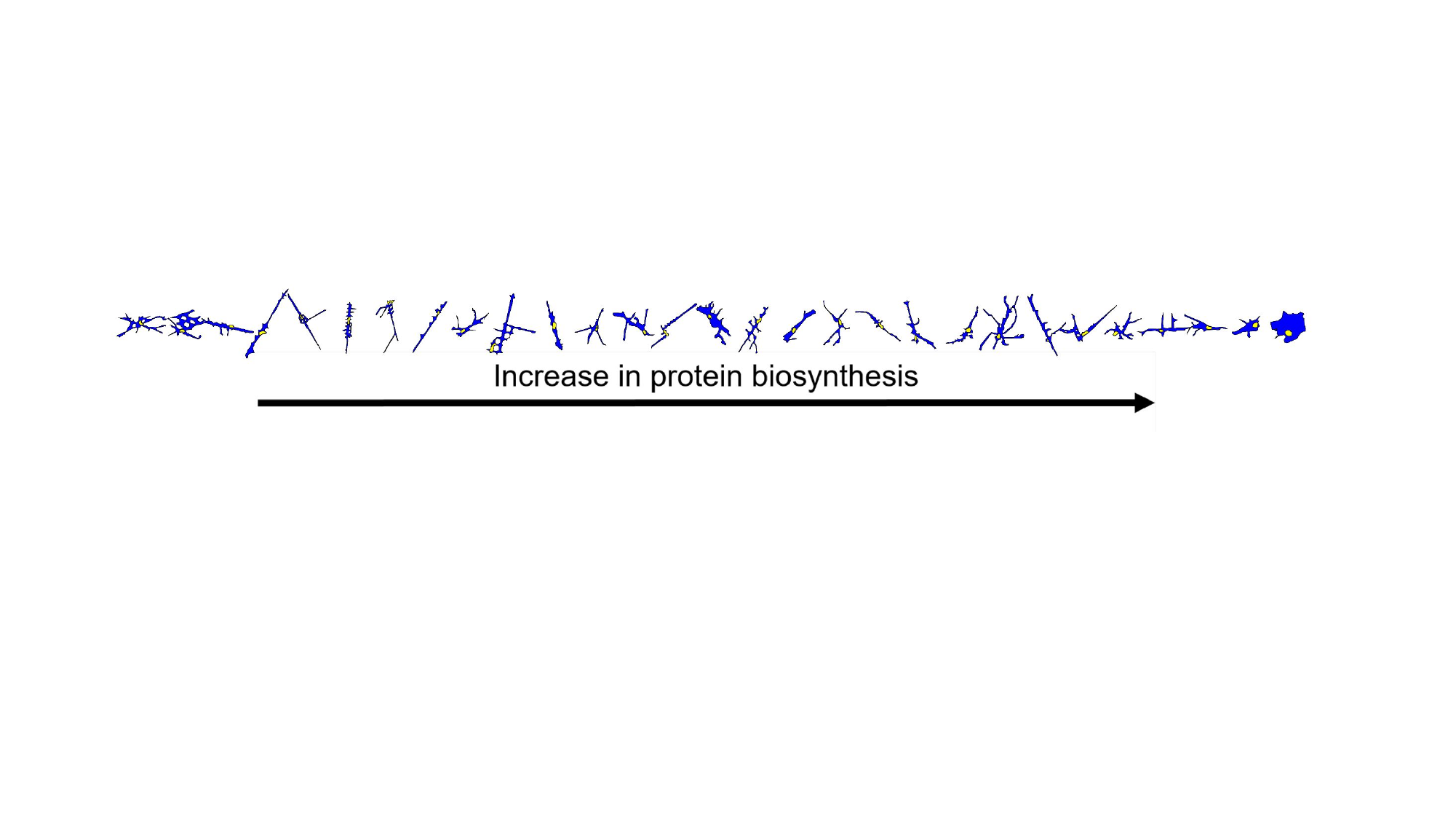

#
